# Preliminary microbiome characterization of shrimp gut and pond water in Egyptian aquaculture farms: Implications for pathogen dynamics and management practices

**DOI:** 10.1007/s11259-026-11113-7

**Published:** 2026-03-30

**Authors:** Eman Zahran, Samia Elbahnaswy, Timothy J. Bruce, Yassmin E. Hegab, Dusan Palic

**Affiliations:** 1 Department of Aquatic Animal Medicine, Faculty of Veterinary Medicine, Mansoura University, Mansoura, 35516 Egypt; 2 Horus Research Center, Horus University-Egypt (HUE), Coastal Road, New Damietta, 34518 Egypt; 3 Academy of Scientific Research & Technology, 101 El Kasr Alaini St, Cairo, 11516 Egypt; 4 School of Fisheries, Aquaculture & Aquatic Sciences, Auburn University, Auburn, AL 36830 USA; 5 Lakes Protection and Fish Resources Development Authority, New Cairo, Cairo, Egypt; 6 Fish Diseases and Fisheries Biology, Faculty of Veterinary Medicine, Ludwig-Maximilians University, Munich, Germany

**Keywords:** Metagenomics, Crustacean, Intestine, Microbial, Sequencing, Aquaculture

## Abstract

Shrimp aquaculture is a rapidly expanding food sector; however, its sustainability is challenged by disease outbreaks often linked to imbalances in the microbiome. Here, we characterized the microbial communities in the intestines of shrimp and pond water from three Egyptian farms (A, B, and C) using Oxford Nanopore long-read sequencing. Descriptive comparisons of relative abundance and diversity trends revealed that pond water harbored significantly higher alpha diversity than shrimp guts. In contrast, beta diversity confirmed a strong separation between host-associated and environmental microbiomes. For the observed phyla, taxonomic profiling revealed that shrimp guts were dominated by Proteobacteria, Actinomycota, and Bacillota, whereas pond water contained additional constituents, including Cyanobacteria and Bacteroidota. Pathogen-associated genera, particularly *Vibrio* spp. and *Pseudomonas* spp., were more abundant in water samples, with farm-specific variations linked to management practices such as salinity and feed protein content. Venn analysis highlighted that pond water harbored the largest pool of unique taxa, reinforcing its role as a putative reservoir for pathogens. These findings provide the first integrative microbiome baseline for Egyptian shrimp farms, underscoring the need for microbiome-informed management to mitigate the risk of pathogens.

**Supplementary Information:**

The online version contains supplementary material available at 10.1007/s11259-026-11113-7.

## Introduction

Aquaculture is a rapidly growing animal food production industry, now supplying approximately 50% of the globe’s seafood (FAO 2020). By 2016, aquaculture had surpassed capture fisheries as the primary source of seafood for human consumption, contributing more than 53% of the total global supply (Bailey and Tran 2019). This transition represents a fundamental shift in global food production systems and has positioned aquaculture as a central pillar of food security strategies worldwide (Tacon and Shumway 2024). Pacific white shrimp (*Litopenaeus vannamei*) is one of the highest quality cultured crustaceans owing to its fast growth, broad salinity tolerance, and high market demand (Azizah and Samaadan 2024; Sadek and Nabawi 2021; Omar et al. 2024; Pratiwi et al. 2021). However, the intensification of shrimp farming systems has been accompanied by persistent disease challenges that continue to limit productivity and profitability (Choudhary et al. 2025; Emerenciano et al. 2022). Bacterial diseases, particularly those caused by opportunistic *Vibrio* species, remain a recurring and seasonally driven threat in shrimp pond culture globally (Ghosh et al. 2021). These disease events often arise from complex interactions among the host, its associated microbiota, and the surrounding aquatic environment (Huang et al. 2021). Acute hepatopancreatic necrosis disease (AHPND), also known as early mortality syndrome (EMS), is one of the most severe bacterial diseases affecting *Litopenaeus vannamei *aquaculture and is primarily caused by virulent strains of *Vibrio parahaemolyticus *carrying the plasmid-encoded PirA and PirB toxins (Lee et al. 2015). Outbreaks of AHPND have resulted in substantial economic losses and have highlighted the importance of microbial community shifts in pond water and shrimp gut as key drivers of *Vibrio *proliferation and disease susceptibility (Zorriehzahra, 2015).

In recent years, increasing attention has been directed toward the role of the microbiome as a key determinant of host health. In shrimp, intestinal microbial communities contribute not only to digestion and nutrient assimilation but also to immune regulation, growth performance, and resistance to pathogen colonization (Angthong et al. 2023; Holt et al. 2021; Kumar et al. 2023). Conversely, microbial dysbiosis within the gut has been associated with increased disease susceptibility and compromised host condition (Xiong 2018). Pond water represents a dynamic microbial reservoir capable of influencing shrimp health through continuous exposure, microbial recruitment, and the dissemination of potential pathogens (Huang et al. 2018). In addition, environmental factors such as salinity further shape microbial assemblages by selecting for distinct bacterial taxa under different physicochemical conditions (Vinay et al. 2023).

Farm-level management practices, including salinity regulation, feeding strategies, stocking density, and pond preparation, play a critical role in structuring both host-associated and environmental microbiomes (Landsman 2019). For example, high-protein feeding regimes can elevate organic loading in ponds, promoting the proliferation of opportunistic bacteria such as *Vibrio* spp., while chemical disinfectants used during pond preparation may disrupt native microbial communities and create niches for resistant taxa (Zoqratt et al. 2018). Collectively, these factors contribute to substantial variability in microbial composition across farms, with direct implications for disease risk and production outcomes (Xiong 2018; Zoqratt et al. 2018).

Although shrimp microbiome studies have been extensively conducted in Asia and Latin America (Huang et al. 2021; Xiong et al. 2017), comparable investigations in North Africa and the Mediterranean region remain limited and have largely focused on species identification or general fisheries biology rather than gut and environmental microbial ecology (Ahmed et al. 2021; Elgendy et al. 2015; Sharawy et al. 2017).

Egypt represents a particularly relevant setting for such work due to its rapidly expanding shrimp aquaculture sector and the pronounced heterogeneity in farming practices across regions (Feidi 2018). Egyptian shrimp farms differ markedly in salinity regimes, pond preparation protocols, feed protein content, and the use of co-culture species (Abou El-Amaiem 2015; El-Damhogy et al. 2016). Despite this variability, there remains limited understanding of how these practices influence microbial diversity and pathogen dynamics within production systems.

Advances in high-throughput sequencing technologies now allow for high-resolution characterization of microbial communities (Sundaray et al. 2022). Long-read sequencing approaches, such as Oxford Nanopore full-length 16 S rRNA sequencing, provide enhanced taxonomic resolution at the genus and species levels compared with short-read methods and facilitate improved detection of ecologically and clinically relevant taxa (Lin et al. 2024; Older et al. 2024). When integrated with farm management data, these approaches offer a powerful framework for linking microbial patterns to environmental and operational variables.

In the present study, we employed full-length 16 S rRNA sequencing using the Oxford Nanopore platform to characterize and compare the gut and pond water microbiomes of *L. vannamei* across three Egyptian shrimp farms with distinct management practices. We assessed alpha and beta diversity, taxonomic composition at the phylum and genus levels, and the occurrence of putative pathogenic taxa. By integrating microbiome data with detailed farm histories, including salinity, feed composition, pond preparation, and co-culture practices, we aimed to evaluate how environmental and management factors shape microbial community dynamics. To our knowledge, this study represents the first multi-farm comparative analysis of shrimp gut and pond water microbiomes in Egyptian aquaculture and provides a foundational framework for future disease risk assessment and the development of sustainable management strategies.

## Materials and methods

### Farm history data collection

Farm management histories were documented for Farms A, B, and C, including salinity levels, pond preparation methods, feed types and protein content, pond size, stocking densities, and species composition. Table 1 lists a complete record of shrimp farm histories during sampling. These contextual data were integrated with microbiome analysis results to explore correlations between management practices and microbial outcomes.

**Table 1:** Characterization of shrimp farms incorporated into the study design

Parameter	Farm A	Farm B	Farm C
Antibiotic usage	No	No	No
Water temperature (°C)	26	25	26
Total pond area (feddan)	9	11	80
Total ponds	3	4	20
Pond Size (feddan)	1.5	1.5	2
Water source & irrigation	Mediterranean Sea (pump)	Mediterranean Sea (pump)	Bahr El-Midan (connected to Boghaz)
Salinity (**‰**, ppt)	50	33	45
Drainage	Gravity	Gravity	Gravity
Fish species onsite	Shrimp, seabream, seabass	Shrimp, seabream, seabass	Shrimp, seabream, seabass, meagre, mullet
No. of shrimp ponds	2	4	5
Culture system	Semi-intensive		
Feed type & protein	Manufactured (30%)	Manufactured (32%)	Manufactured (38%–44%)
Feeding method & frequency	Hand feed, twice daily		
Shrimp size at harvest (pieces/kg)	70	60	50
Shrimp age at harvest (months)	12	8	12

### Sample collection

**Fig. 1:**
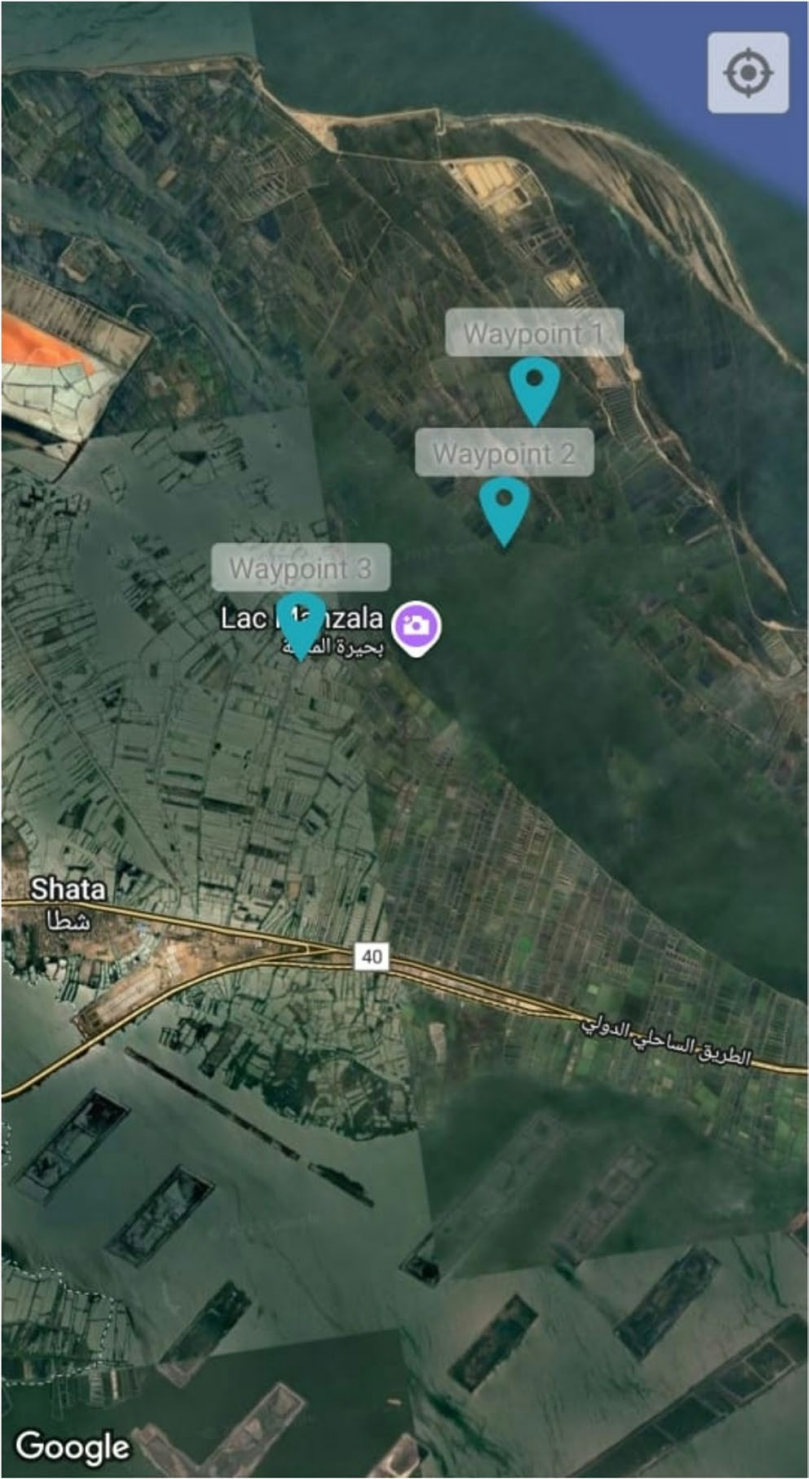
A map from google earth showing farm sites in Damietta, Egypt. waypoint 1–3 correspond to farms A–C, respectively

Shrimp (*L. vannamei*) were collected from a private farm in the Shatta area, Damietta Governorate, Egypt. Figure 1 shows the locations of each farm. A total of 15 shrimps from each farm, independent of the total number of ponds, were collected, with an average of 15–20 g. Shrimp were sampled from representative ponds reflecting the typical farm conditions. For the microbiome study, water was collected using 500 ml sterile disposable plastic bottles. Triplicate samples were collected from each farm. The triplicate samples from each farm were bulked and homogenized in equal portions (50 mL of water sample). Water samples were subjected to a series of centrifugations at ~ 1,000 × g for 5–10 min. Supernatants were aliquoted and centrifuged again at 10,000 × g for 20 min. Pellets were then resuspended in PBS and stored at − 80 °C for downstream processing (Mahmoud and Magdy 2021). Gut samples were dissected under aseptic conditions, preserved on dry ice, and stored at − 80 °C until DNA extraction.

### DNA extraction and library preparation

Following the manufacturer’s instructions, DNA was extracted from the shrimp guts and farm water samples using the Sherlock AX DNA Extraction Kit (A&A Biotechnology, Gdansk, Poland). The DNA concentrations were checked using a Qubit™ 4 Fluorometer (Thermo Fisher Scientific, Waltham, MA, USA). The Native Barcoding V14 kit (Oxford Nanopore Technologies, UK) with Long Fragment Buffer (LFB) following the manufacturer’s recommendations was used for the preparation of the libraries (Oxford Nanopore Technologies, UK). Sequencing was performed, using a MinION sequencer (MIN-101B; Oxford Nanopore Technologies, UK) with a 10.4.1 Flow cell (FLO-MIN114; Oxford Nanopore Technologies, UK).

### Metagenomic sequencing and data analysis

Raw and processed FASTA files generated in this study have been deposited in Zenodo (DOI: 10.5281/zenodo.17915315). Sequences were first analyzed using the Galaxy Europe web platform (Galaxy). The assessment of read quality before and after preprocessing was performed using FastQC, and Nanoplot (Ewels et al. 2016). The trimming and filtering of reads by length and quality were conducted using Porechop (Afgan et al. 2018). Kraken2 was then used for database classification. For visualization of the results, the R program (v4.2.3) with ggplot2 were incorporated. Diversity indices (Observed, Chao1, ACE, Shannon, Simpson, InvSimpson, and Fischer) were calculated in R using the phyloseq, vegan and tidyverse packages. A Venn diagram was generated using the VennDiagram package to assess shared and unique taxa.

### Alternative alpha diversity analysis of shrimp intestines with host depletion

To attain additional insight into the alpha diversity metrics from the samples, an additional analysis was performed using host depletion. Briefly, raw read fastq files of the shrimp gut and water samples were organized into Epi2Me (Oxford Nanopore Technologies). The wf-metagenomics workflow (v 2.13.0) was implemented for analysis. For parameters, the *L. vannamei* genome (NCBI RefSeq Assembly GCF_042767895.1) was depleted from the analysis, and Kraken2 (v. 2.1.3) was used for read classification. The output alpha diversity parameters for the shrimp guts only were analyzed using GraphPad Prism (v 10.5.0), where a one-way ANOVA was performed, following an assessment of model residuals with the Shapiro-Wilk test and variance equivalence with a Brown-Forsythe test. If the assumptions for each parameter were not met, a Kruskal-Wallis nonparametric analysis was performed.

## Results

### General microbial profile

High-throughput shotgun metagenomic sequencing generated a total of 1,660,152 reads across all shrimp gut and pond water samples. After quality filtering and host depletion, 1,023,599 reads (61.65%) were retained for downstream taxonomic classification, while 435,056 reads (26.2%) remained unclassified or unmapped. Read retention varied between sample types. Shrimp gut samples retained between 33.65% and 68.03% of reads after host depletion, reflecting the presence of host-derived DNA. However, pond water samples exhibited consistently high retention rates (98.36–99.35%), indicating effective enrichment of microbial sequences. Taxonomic classification demonstrated that most classified reads belonged to the bacterial domain. Across all samples, a diverse microbial community was detected, comprising taxa from multiple phyla. The dominant phyla were Proteobacteria, Bacteroidota, Actinomycetota, Cyanobacteriota, and Firmicutes, accounting for the majority of classified sequences (Table **S1**).

### Metagenomic sequencing analysis

#### Alpha diversity

**Fig. 2:**
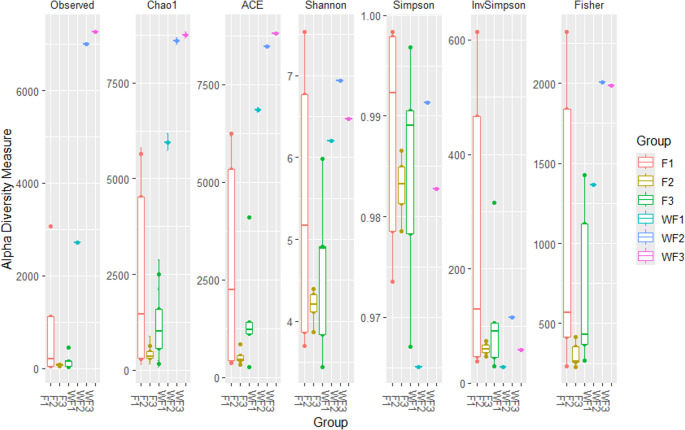
Alpha diversity metrics of shrimp and water samples at the three shrimp farms F1-3 = shrimp farms A-C, WF1-3 = WF A-C, water samples

Shannon and Simpson indices showed that pond water harbored higher microbial diversity and evenness compared to shrimp guts (median Shannon: 1.01 vs. 0.70). Non-parametric Wilcoxon tests suggested greater richness in water (*p* = 0.404, exploratory), with effect sizes indicating moderate differences. Simpson diversity was lower in water (0.27 vs. 0.50, *p* = 0.072), reflecting higher community evenness. No significant farm-to-farm differences were detected in shrimp samples (Kruskal–Wallis, *p* > 0.05) **(**Fig. 2**)**.

#### Beta diversity

**Fig. 3:**
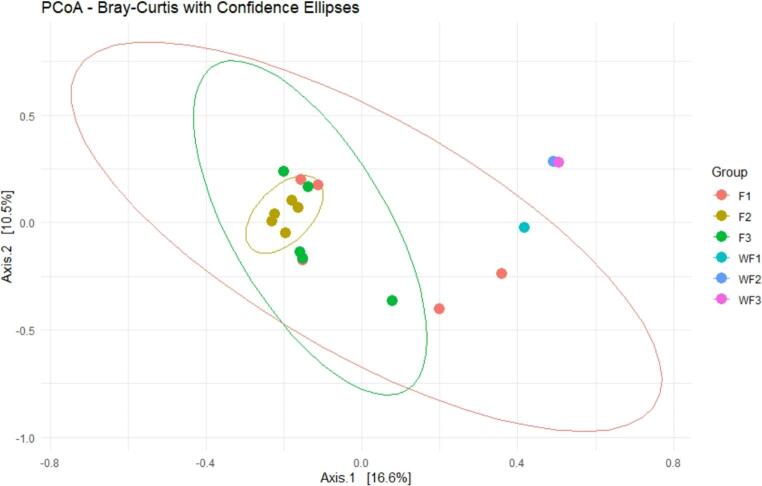
Principal Coordinates Analysis (PCoA) plot summarizing the differences in microbial community composition (beta diversity) among six groups using the Bray–Curtis dissimilarity metric. F1-3 = shrimp farms A-C, WF1-3 = WF A-C, water samples

Bray–Curtis PERMANOVA demonstrated a strong, significant separation between shrimp gut and pond water microbiomes (pseudo-F = 36.01, R² = 0.679, *p* = 0.0015). Clear separation Between Water (WF) and Gut (F) Samples, where the three WF groups (WFA–C) are positioned far from the F groups (FA–C) on the PCoA space, particularly along Axis 1, indicating substantial compositional differences. Low Intra-Group Variation in FB, where samples in FB (yellow) cluster tightly within a small ellipse, reflecting low variability and a stable, consistent microbial community within this group. In contrast, there is a moderate Spread in FA and FC, where FA (red) and FC (green) display larger ellipses, suggesting greater inter-individual variation among shrimp gut microbiomes in those groups. Pond water samples form distinct, isolated Points, where WFB and WFC (blue and pink) are clearly separated from each other and from the gut groups, indicating that even among water samples, microbial profiles differ across locations or tanks. PCoA plots illustrated the tight clustering of shrimp gut samples and dispersed water samples **(**Fig. 3**).**

#### Taxonomic composition

**Fig. 4:**
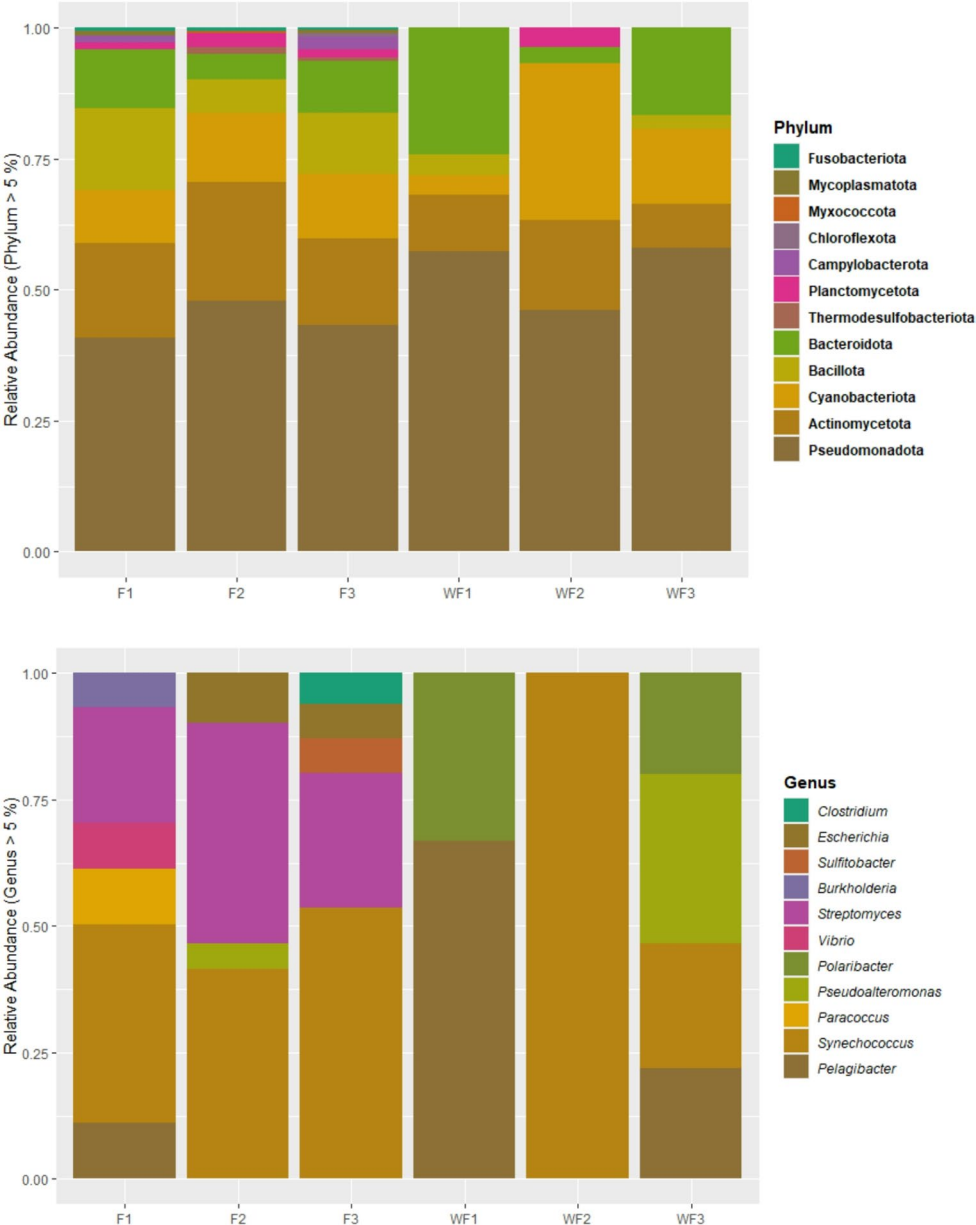
Relative abundance of identified constituents, by phyla and genera. F1-3 = shrimp farms A-C, WF1-3 = WF A-C, water samples

**Fig. 5:**
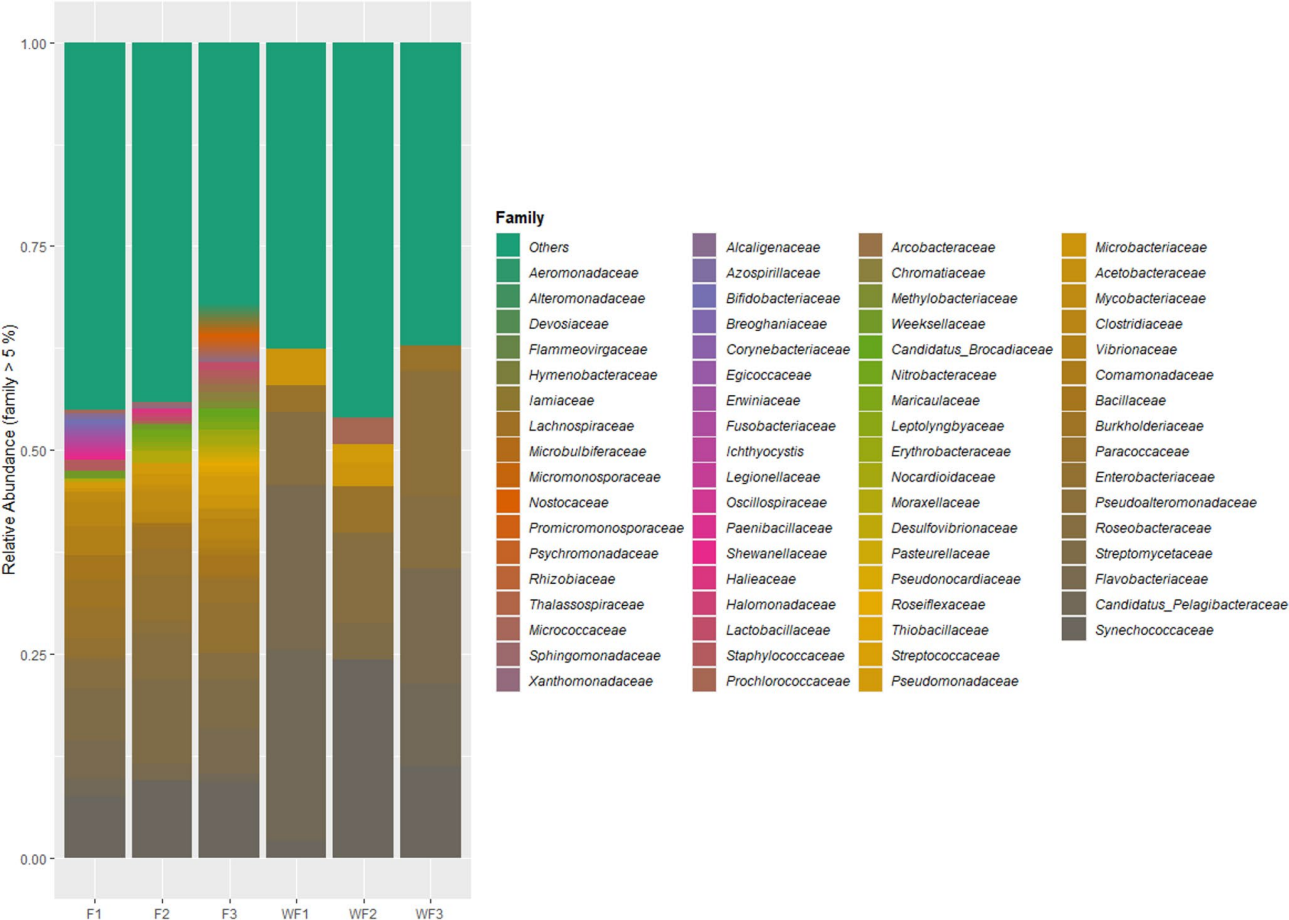
Family-level relative abundance of shrimp and water samples from the respective Egyptian shrimp farms. F1-3 = shrimp farms A-C, WF1-3 = WF A-C, water samples

Taxonomic profiling revealed clear differences between shrimp gut (F1–F3) and pond water (WF1–WF3) microbial communities across phylum, genus, and family levels **(**Figs. 4 and 5**)**. The phylum-level composition reveals clear differences between shrimp intestines (FA–C) and surrounding farm water (WFA–C). Pseudomonadota dominated both environments and was particularly enriched in WFB and WFC. Actinomycetota, Bacillota, and Cyanobacteriota were present in both sample types but showed a more balanced relative abundance in shrimp gut samples compared with pond water. Bacteroidota exhibited higher relative abundance in WFA. Several low-abundance phyla, including Myxococcota, Campylobacterota, and Planctomycetota, were detected mainly in shrimp gut samples and occurred only at very low abundance in water. At the genus level, pronounced differences were observed between host-associated (F) and environmental (WF) communities. Paracoccus and Streptomyces showed higher relative abundance in shrimp gut samples, whereas Pelagibacter was strongly enriched in WFA and WFC. Polaribacter was more abundant in WFA and WFC and is typically associated with marine particles and algal-derived organic matter. Genera such as Escherichia, Sulfitobacter, and Vibrio occurred sporadically in both shrimp gut and pond water samples at low to moderate relative abundance. Family-level profiles were consistent with patterns observed at higher taxonomic ranks. Streptomycetaceae, Micrococcaceae, and Paracoccaceae were relatively more abundant in shrimp gut samples, while Pelagibacteraceae, Flavobacteriaceae, and Vibrionaceae were more dominant in pond water samples. A large proportion of sequences across all samples belonged to the “Others” category, particularly in shrimp gut samples, indicating greater taxonomic complexity in the intestinal microbiota than in pond water communities.

#### Pathogen-associated genera

**Fig. 6:**
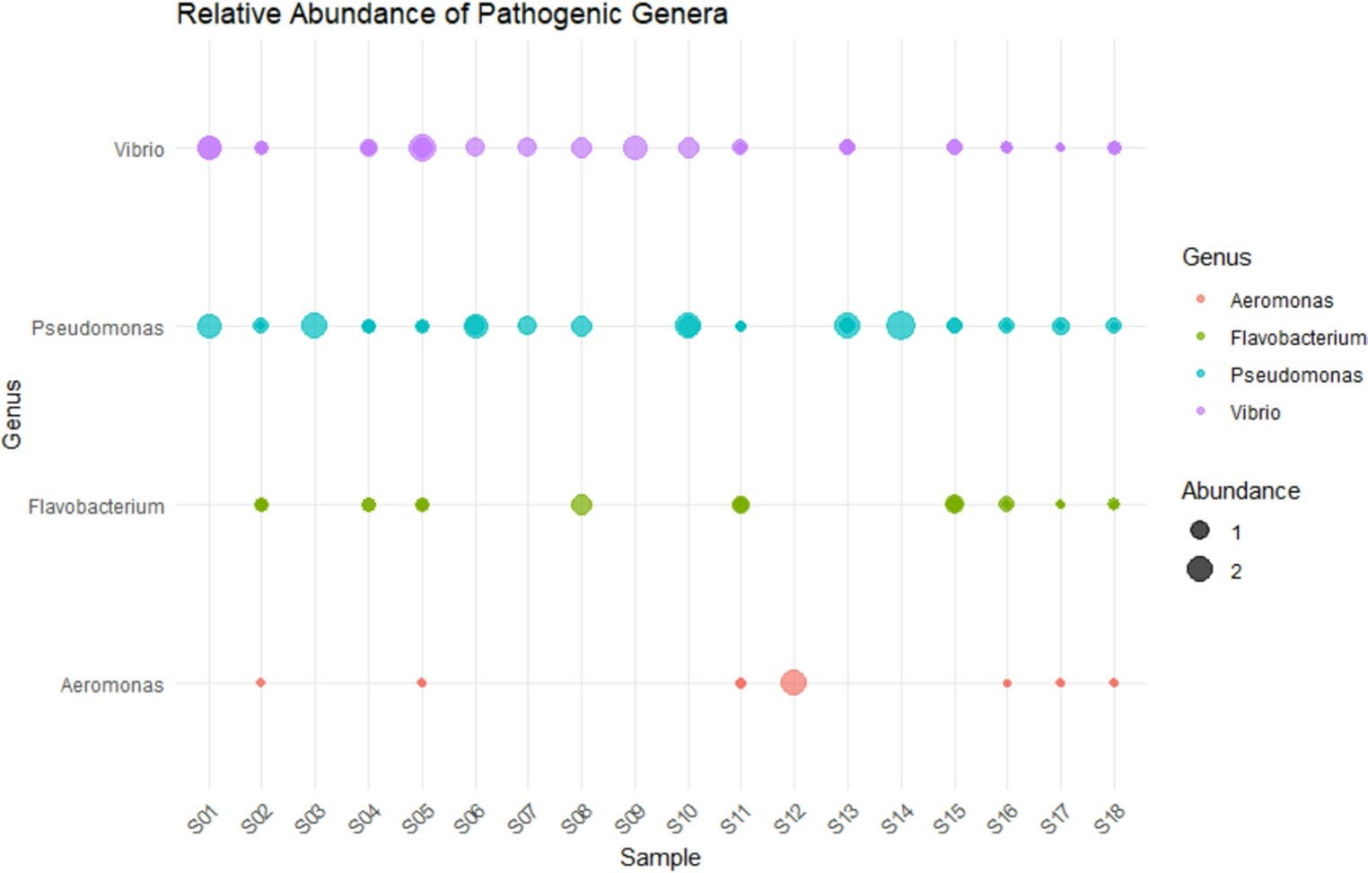
Relative abundance of pathogenic genera detected in shrimp gut and water samples at three shrimp farms. S1-15 = shrimp farms (*n* = 5/farm), S16-18 = water samples

The relative abundance of selected potentially pathogenic genera is shown in Fig. 6. Among the detected taxa, *Vibrio* exhibited the widest distribution and was frequently observed in shrimp gut and water samples from all farms, indicating its broad occurrence across farms and sample types. *Pseudomonas* was detected in most samples but showed higher relative abundance in shrimp gut samples, particularly FA (S01–S05) and FB (S06–S10), suggesting that *Pseudomonas *may be more host-associated, or enriched in the gut environment. *Flavobacterium* was present in nearly all samples, most pronounced in FA and FB samples (S07–S15), but only at low relative abundance. Its distribution suggests *Flavobacterium* may be influenced by host conditions or environment-specific factors. *Aeromonas* was the least abundant and most sporadically detected genus. It was detected in a low level in FA (S01, S03), slight presence in FB (S06), and noticeably higher abundance in FC (S12). In contrast, it showed a very low levels in all pond water samples. The spike in S12 (FC) may indicate early-stage infection, host-specific dysbiosis, or exposure to contaminated water.

#### Community overlap (Venn analysis)

**Fig. 7:**
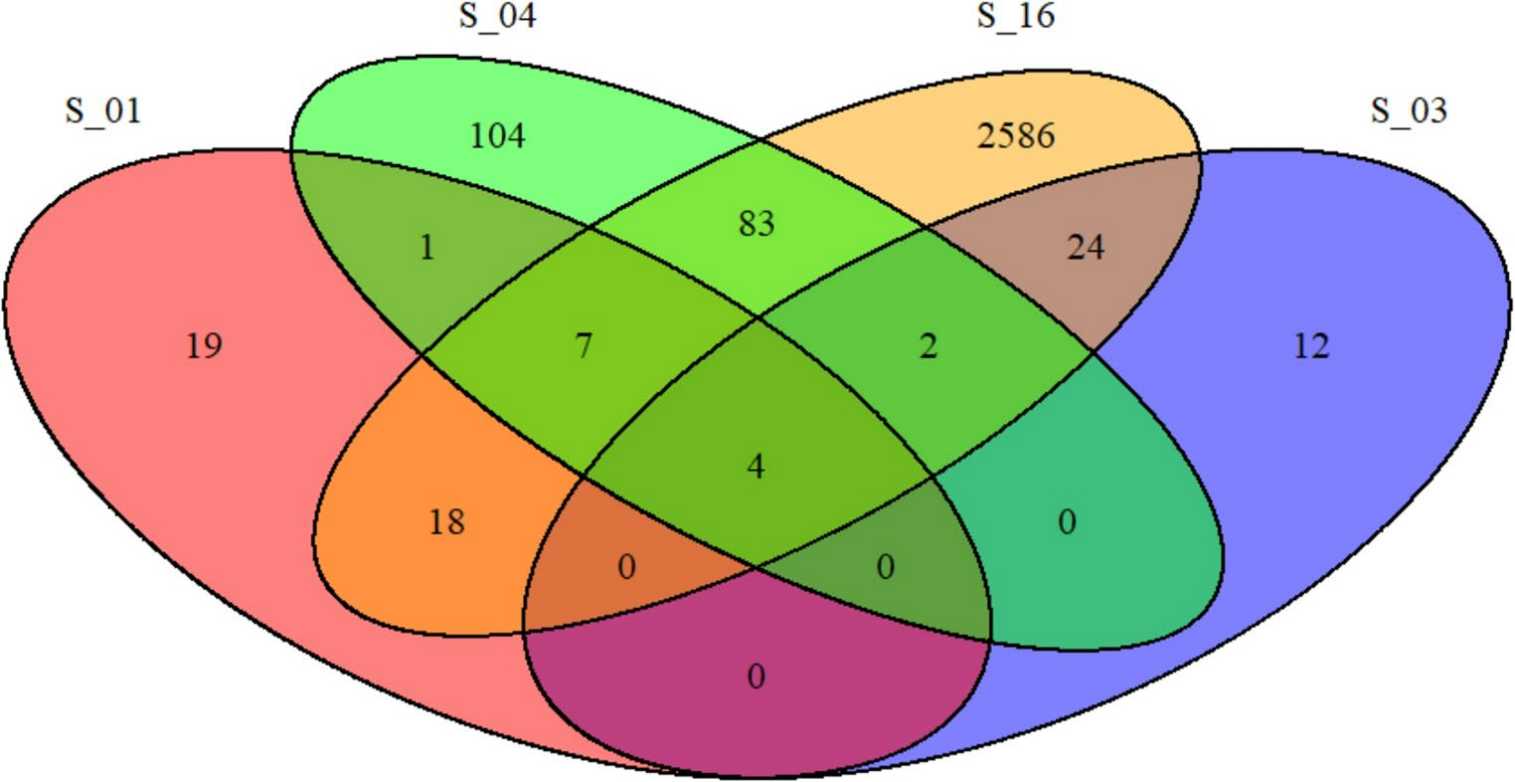
Venn diagram visualizes the overlap and uniqueness of taxonomic features (e.g., ASVs or OTUs) across the four samples: Farm A: S_01, S_03, S_04 (shrimp gut), and S_16 (water sample). Each ellipse represents one sample, and the numbers indicate how many taxa are unique to one sample or shared among multiple samples

The Venn diagrams showed a small set of phyla shared across shrimp and water samples, while most taxa were habitat-specific. Water samples carried the largest number of unique phyla, particularly in Farms B and C. Shrimp guts contributed fewer unique taxa, with Actinomycetota consistently shared across environments **(**Fig. 7**)**.

### Alpha diversity of gut samples with host depletion

**Fig. 8:**
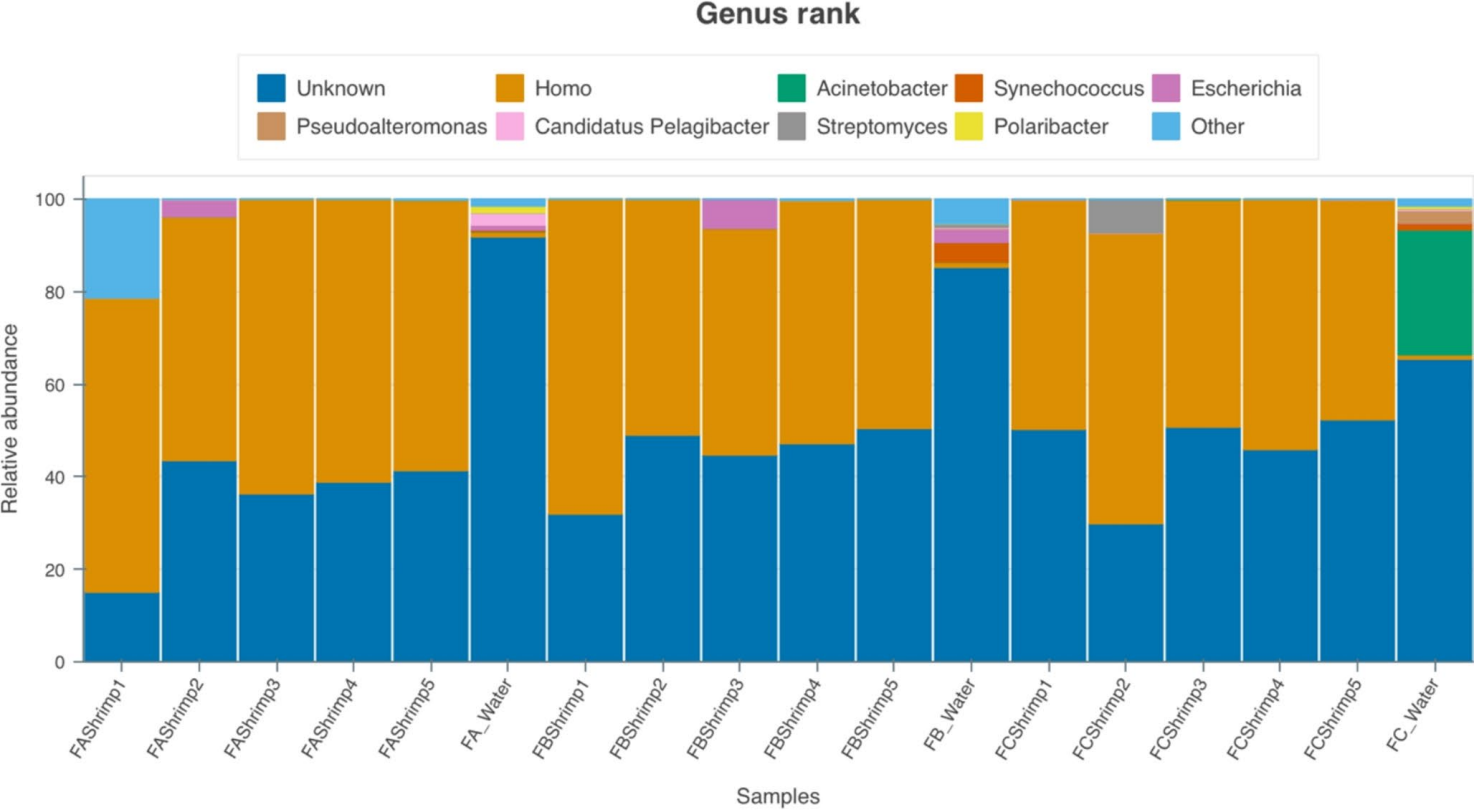
Bar plot of relative abundance of shrimp and gut water samples following host depletion (*L. vannamei*). FA=Farm A; FB=Farm B; FC=Farm C; and 1–5 denote individual gut samples from five shrimp per location

Following the host depletion, 98.8% of reads in the water sample remained, while an average of 58.0% of reads remained for the intestinal samples. The outcomes from the alpha diversity calculations for the shrimp gut samples are summarized in Table 2. No differences were detected across the farms in terms of alpha diversity. Similar to the primary analysis, the overall relative abundance of the intestinal and water samples differed significantly, as depicted in Fig. 8. All samples analyzed, including water samples, had a large composition of human-related taxa. Of note were instances of some higher abundances of *Escherichia* spp. in individual shrimp from Farms A and B. A single sample at Farm C also had an elevated relative abundance of *Streptomyces* spp. Overall, although host depletion removed a substantial number of host-related taxa, the additional analysis did not provide enhanced resolution in the alpha diversity analyses.

**Table 2:** Alpha diversity metrics of shrimp intestinal samples following *L. vannamei* host depletion

Parameter	Farm A	Farm B	Farm C	*P*-value
Effective species number	2.13±0.11	2.06±0.09	2.07±0.06	0.849
Fisher’s alpha	84±18	93±11	105±31	0.803
Shannon diversity	0.75±0.05	0.72±0.04	0.73±0.03	0.880
Simpson’s index	0.50±0.14	0.50±0.02	0.50±0.00	0.979
Pielou’s evenness	0.69±0.14	0.87±0.07	0.65±0.11	0.367
Richness	5.0±1.8	2.4±0.2	3.8±0.9	0.308

## Discussion

The present study demonstrates clear differentiation between shrimp gut and pond water microbiomes, supporting the hypothesis that microbial community structure is strongly governed by ecological niche. While pond water harbored diverse and environmentally driven microbial assemblages, the shrimp gut microbiome exhibited lower diversity and greater compositional consistency, suggesting strong host-mediated selection.

Alpha diversity analyses indicated that pond water communities possessed higher richness and evenness than shrimp gut communities, as reflected by Shannon and Simpson indices. This pattern is consistent with previous studies in shrimp aquaculture systems, which have repeatedly shown that rearing waters support broader microbial diversity than host-associated niches For example, Zoqratt et al. (2018) showed that pond water microbiota were more diverse than shrimp gut microbiota, including only some main principle taxa, such as Vibrio and Photobacterium, and suggested host-specifying microbial choice. Guo-Rong et al. (2018) demonstrated higher alpha diversity in pond water compared to the shrimp gut. Our data validates this, displaying a more evenly spread microbial community in pond water, and a larger, more specific-tone microbiota in shrimp guts.

Bray–Curtis dissimilarity-based beta diversity and PERMANOVA analysis revealed significant differentiation between pond shrimp and shrimp gut microbiomes. This finding is consistent with earlier studies that have shown microbial community composition to be primarily determined by habitat. According to Rajonhson et al. (2024), Similar habitat-driven clustering has been reported in prior investigations comparing shrimp gut, sediment, and water microbiomes, where ecological niches account for more than half of the observed variability. In the same context, separate microbial community clusters have also been reported for the shrimp gut, pond water, and sediment, based on 16 S rRNA gene sequencing and beta diversity measures (Lalitha et al. 2023; Liu et al. 2022). The tight clustering of shrimp gut samples observed here suggests a relatively stable and resilient intestinal microbiome, potentially maintained through host immune regulation and competitive exclusion mechanisms.

Taxonomic profiling further highlighted contrasts between environments. Shrimp gut samples were dominated by Proteobacteria, Actinomycetota, and Bacillota, whereas pond water samples contained additional phyla, including Cyanobacteriota and Bacteroidota, particularly in Farms B and C. This distinction reflects the heterogeneous and environmentally responsive nature of pond microbial communities compared with the filtered microbial assemblage of the shrimp gut (Amin et al. 2024; Huang et al. 2018). Our results coincided with a prior study, exhibiting enriched shrimp gut microbiota in Rhodobacteraceae and Actinobacteria, whereas pond samples had more Vibrionaceae, Firmicutes, and Cyanobacteria (Landsman et al. 2019). Moreover, the presence of *Cetobacterium* and *Rhodococcus* in the shrimp gut suggests potential roles in vitamin biosynthesis and lipid metabolism, as proposed earlier in host–microbiome interaction studies. These findings reinforce the concept that host-associated microbiomes are shaped by selective pressures distinct from those operating in the surrounding environment.

Potentially pathogenic genera, such as *Vibrio*, *Pseudomonas*, and *Flavobacterium*, were more frequently detected in shrimp gut samples than pond water, underscoring the importance of pond water as a source for opportunistic pathogens. Our results align with those of Yu et al. (2022) and Beltrán et al. (2023), who linked increased *Vibrio* abundance in pond water to disease outbreaks, including acute hepatopancreatic necrosis disease (AHPND) and translucent post-larval disease. The relatively low prevalence of these taxa within shrimp guts in the present study suggests effective host-mediated exclusion under non-stressed conditions. Nevertheless, the presence of such pathogens in the environment represents a latent risk, particularly under conditions of immune suppression or environmental stress.

Venn diagram analyses revealed limited taxonomic overlap between gut and water samples, with most taxa exhibiting habitat specificity. Pond water samples contained the largest number of unique phyla, particularly in farms with higher cyanobacterial representation. These results are consistent with those of Liu et al. (2022), who found that shrimp acquire a high percentage of their gut microbiota from inland water as the farming procedure continues. Likewise, Zoqratt et al. (2018), observed that while environmental microbiota contribute to gut colonization over time, the shrimp gut microbiome remains compositionally distinct due to selective recruitment processes.

Host DNA depletion strategy successfully reduced host-derived reads, particularly in gut samples, although the approach did not substantially alter alpha diversity outcomes. Approximately 58% of microbial reads were retained in intestinal samples following depletion, reflecting the persistent challenge of host DNA contamination in gut microbiome studies. The lack of enhanced diversity resolution following depletion suggests that sequencing depth alone may not fully capture low-abundance taxa or overcome upstream limitations such as contamination or methodological bias (Cheng 2021; Kim et al. 2024).

Notably, the detection of human-associated taxa such as *Escherichia* spp. and *Streptomyces* spp. in certain samples raises the possibility of anthropogenic influence or environmental contamination within aquaculture systems. Similar observations have been reported in other microbiome studies and highlight the importance of rigorous quality control and contamination-aware analytical workflows (Ekanayake et al. 2023). The enrichment of *Escherichia* spp. in individual shrimp from Farms A and B, may reflect transient colonization under high-density or stress-prone conditions (Bruggeling et al. 2020), warranting further investigation.

It is alarming that the incidence of potentially pathogenic genera, such as *Vibrio*, *Photobacterium*, and *Pseudomonas*, in pond samples is high, as these have been associated with diseases among shrimp farms (Dhanush et al. 2025; Murugan et al. 2024). The low overlap between community membership of ponds and guts, as predicted by Venn analysis, also evidenced the selective nature of host colonization, as well as the screening effect that the gut environment imposes on the attacking microbes. While host-mediated selection appears effective under stable conditions, environmental reservoirs of opportunistic pathogens remain a critical factor in disease emergence, consistent with ecological models of microbiome plasticity and host–environment interaction as reported by Chen et al. (2017).

## Conclusion

Overall, these results confirm the hypothesis that pond and shrimp gut microbiomes are host-shaped and low in diversity, while pondwater communities are high in diversity and environmentally determined. The distinct separation between the two environments reinforces the rationale for regular supervision of pond microbiota in the control of disease risk and shrimp health. It will be beneficial in the future to investigate the functional significance of these microbial differences and the potential of microbiome manipulation strategies (e.g., environmental bioaugmentation or probiotics) as a means to enhance sustainability in aquaculture.

## Supplementary information

Below is the link to the electronic supplementary material.

Supplementary File 1 (DOCX 18.4 KB)

## Data Availability

The sequencing data supporting this study are available in the Zenodo repository under the title “Shrimp Microbiome Sequencing Dataset (FASTA Files)”, accessible via DOI: 10.5281/zenodo.17915314.
