# Supplementary material for: Preliminary microbiome characterization of shrimp gut and pond water in Egyptian aquaculture farms: Implications for pathogen dynamics and management practices
Source: Vet Res Commun. 2026 Mar 30;50(3):244. doi: 10.1007/s11259-026-11113-7 (PMC13035673; doi:10.1007/s11259-026-11113-7)
Supplement: Supplementary file 1 — (DOCX 18.4 KB) [file 11259_2026_11113_MOESM1_ESM.docx]

| **[index](file:///C:\\Users\\Startklar\\Downloads\\wf-metagenomics-report%20(1).html)** | [**Reads**](file:///C:\Users\Startklar\Downloads\wf-metagenomics-report%20(1).html) | [**Reads after host depletion**](file:///C:\Users\Startklar\Downloads\wf-metagenomics-report%20(1).html) | [**Unclassified\|Unmapped**](file:///C:\Users\Startklar\Downloads\wf-metagenomics-report%20(1).html) | [**Reads after host depletion (%)**](file:///C:\Users\Startklar\Downloads\wf-metagenomics-report%20(1).html) | [**Unclassified\|Unmapped (%)**](file:///C:\Users\Startklar\Downloads\wf-metagenomics-report%20(1).html) |
| --- | --- | --- | --- | --- | --- |
| **FAShrimp1** | 7092 | 4650 | 695 | 65.57 | 9.8 |
| **FAShrimp2** | 237099 | 103499 | 44992 | 43.65 | 18.98 |
| **FAShrimp3** | 3255 | 2066 | 746 | 63.47 | 22.92 |
| **FAShrimp4** | 25501 | 14082 | 5457 | 55.22 | 21.4 |
| **FAShrimp5** | 542134 | 182451 | 75324 | 33.65 | 13.89 |
| **FA_Water** | 35747 | 35162 | 30868 | 98.36 | 86.35 |
| **FBShrimp1** | 12949 | 8531 | 2718 | 65.88 | 20.99 |
| **FBShrimp2** | 30165 | 20521 | 10045 | 68.03 | 33.3 |
| **FBShrimp3** | 3890 | 2372 | 1044 | 60.98 | 26.84 |
| **FBShrimp4** | 9536 | 5768 | 2708 | 60.49 | 28.4 |
| **FBShrimp5** | 16237 | 10723 | 5396 | 66.04 | 33.23 |
| **FB_Water** | 254101 | 252453 | 205177 | 99.35 | 80.75 |
| **FCShrimp1** | 240494 | 107860 | 54185 | 44.85 | 22.53 |
| **FCShrimp2** | 4553 | 2771 | 818 | 60.86 | 17.97 |
| **FCShrimp3** | 39038 | 23799 | 12061 | 60.96 | 30.9 |
| **FCShrimp4** | 1950 | 1130 | 511 | 57.95 | 26.21 |
| **FCShrimp5** | 52421 | 32992 | 17250 | 62.94 | 32.91 |
| **FC_Water** | 372155 | 367069 | 237345 | 98.63 | 63.78 |

**Table S1.** Number of reads after applying read length and quality filters.

Read counts will also reflect host depletion and unclassified reads (kraken2 approach) and unmapped reads (minimap2 approach). Percentages are calculated from reads after the filtering.
